# A large-scale study integrating CEA and tumor deposits to evaluate prognosis in colon cancer patients

**DOI:** 10.3389/fonc.2025.1626538

**Published:** 2025-12-17

**Authors:** Ya’nan Fan, Yun Sun, Xiaojie Hu, Lifei Zhang, Jiantao Dong, Fei Liu, Zhiqiang Wang, Jianhui Cai

**Affiliations:** 1 Department of Surgery, Hebei Medical University, Shijiazhuang, Hebei, China; 2 Department of Gastrointestinal Surgery, Hebei General Hospital, Shijiazhuang, Hebei, China; 3 Department of General Surgery, Shijiazhuang People's Hospital, Shijiazhuang, Hebei, China; 4 Department of Oncology and Immunotherapy, Hebei General Hospital, Shijiazhuang, Hebei, China; 5 Department of Research and Development (R&D), Yetem Biotech Corp. Ltd., Shijiazhuang, Hebei, China

**Keywords:** colon cancer, carcinoembryonic antigen, tumor deposits, prognostic model, nomogram

## Abstract

**Background:**

Reliable prediction of long-term survival in colon cancer remains limited by staging system constraints. CEA and tumor deposits (TD) are known prognostic markers, but their combined predictive value is underexplored.

**Materials and Methods:**

We retrospectively analyzed 1, 029 patients with locally advanced colon cancer who underwent radical resection. A CEA-TD scoring system was developed and integrated with key clinicopathologic variables to construct a prognostic nomogram.

**Results:**

Both TD(+) and elevated CEA levels were independently associated with significantly worse overall survival (HR for TD = 1.985, CEA = 2.209; all P < 0.01). The CEA-TD score effectively stratified patients into four risk categories, each with distinct survival outcomes (P < 0.001). The final nomogram, incorporating CEA-TD score, T stage, N stage, grade, and tumor location, demonstrated high predictive performance, with AUCs of 0.796, 0.834, and 0.807 for 1-, 3-, and 5-year OS in the training cohort, and comparable values in internal and external validations. The C-index reached 0.800, outperforming traditional prognostic factors. Kaplan-Meier and risk curve analyses confirmed the model’s discriminative capacity.

**Conclusions:**

The CEA-TD-based nomogram offers accurate, clinically applicable risk stratification for LOCC patients, supporting personalized treatment strategies and improved prognostic assessment.

## Introduction

Colorectal cancer is one of the leading causes of cancer-related morbidity and mortality worldwide, with a high incidence and poor prognosis in patients diagnosed at advanced stages (1, 2). The prognosis of CRC patients varies significantly depending on multiple factors, including tumor stage, histological grade, and the presence of distant metastasis (3–5). Despite advancements in surgical techniques, chemotherapy, and targeted therapies, predicting the long-term survival of CRC patients remains a challenge due to the complex nature of the disease and the heterogeneity observed in patient outcomes (6–9). Accurate prognostic models are essential for guiding clinical decision-making and tailoring individualized treatment strategies, particularly in determining the best approach for managing patients with locally advanced colon cancer (LOCC) (10, 11).

Carcinoembryonic antigen (CEA) has long been used as a serum biomarker for CRC, particularly in monitoring disease recurrence and assessing treatment response (12, 13). However, its diagnostic and prognostic accuracy is limited when used alone, especially in patients with early-stage or localized disease. Tumor deposits, defined as clusters of cancer cells outside the primary tumor site, have recently emerged as an important prognostic factor in CRC (14–16). These deposits are associated with poorer outcomes and may reflect aggressive tumor behavior. While both CEA and TD have shown prognostic value individually, combining these two factors has not been fully explored in large-scale studies. The integration of CEA and TD, along with other clinical and pathological variables, may enhance the accuracy of prognostic predictions for CRC patients, providing a more comprehensive tool for clinical decision-making.

The purpose of this study is to address the gap in current research by developing a robust prognostic model for colon cancer patients that integrates CEA and TD with other significant clinical variables. Previous studies have primarily focused on individual markers like TNM staging or CEA levels, but few have investigated the combined impact of CEA and TD on long-term survival outcomes (17–20). We hypothesize that combining CEA and TD with established factors such as T and N stages, histological grade, and tumor size will yield a more accurate prognostic model. By combining these factors, patients could achieve improved precision in risk stratification and benefit from a more personalized approach to treatment and follow-up care.

The present study assessed the prognostic value of the CEA-TD scoring system in locally advanced colon cancer and developed and validated a predictive nomogram incorporating this score.

## Materials and methods

### Patient selection

We included patients who underwent radical colorectal cancer resection at Hebei Provincial People’s Hospital and Shijiazhuang People’s Hospital between January 2010 and December 2020. The inclusion criteria were as follows: (1) post-operative pathological diagnosis of colon adenocarcinoma; (2) age greater than 18 years at the time of diagnosis. The exclusion criteria were as follows: (1) history of any previous cancer or distant metastasis; (2) failure to undergo radical resection for any reason; (3) missing essential clinical or follow-up information (e.g., gender, age at diagnosis, tumor size, TNM stage, or CEA levels); (4) other histological types of tumors; (5) prior neoadjuvant chemotherapy or radiotherapy; (6) post-operative survival time of less than one month; (7) tumors located at the rectosigmoid junction or the rectum.

### Data collection

Clinical and pathological data were extracted from the hospital’s electronic medical records, including various variables such as patient age, gender, marital status, Carcinoembryonic antigen, TNM stage, T stage, N stage, histological grade, maximum tumor diameter, tumor location, number of tumor deposits, survival time, date of death, and survival status. Postoperative pathology results were independently reviewed by two experienced pathologists. In cases of diagnostic discrepancies, a third senior pathologist reviewed the samples. A definitive diagnosis was established when at least two pathologists reached a consensus. Right-sided colon cancer was defined as tumors originating in the cecum, ascending colon, hepatic flexure, or transverse colon, while left-sided colon cancer was defined as tumors originating in the splenic flexure, descending colon, or sigmoid colon. Patient TNM staging was determined according to the eighth edition of the UICC (Union for International Cancer Control) TNM classification for malignant tumors. The primary endpoint of this study was overall survival (OS). Overall survival time was calculated from the date of surgery until the date of death or last follow-up. Death was treated as events in the analysis.

### Prognostic feature screening

In this study, univariate Cox regression analysis was performed using the “survival” package in R software to assess the relationship between clinical-pathological variables and patient survival time in the training set. The clinical-pathological variables included age, gender, marital status, T stage, N stage, TNM stage, histological grade, primary tumor location, tumor size, TD, and CEA levels. Variables with statistical significance were then included in a multivariate Cox regression analysis to calculate the hazard ratios (HR) and their corresponding 95% confidence intervals (CI). The Cox multivariate analysis was used to select statistically significant predictors.

### Construction of CEA-TD scoring

Tumor deposits refer to focal aggregates of tumor cells in the pericolic or perirectal mesenteric fat, which are distinct from the primary tumor and not associated with a lymph node. Histological slides and reports were reviewed to collect the following data. TD(-) indicates the absence of tumor deposits, while TD(+) indicates the presence of one or more tumor deposits. Patients were grouped by CEA status as follows: (1) patients with preoperative CEA ≤ 5.0 ng/mL (CEA(-)); (2) patients with preoperative CEA > 5.0 ng/mL (CEA(+)). A CEA-TD scoring system was developed based on the expression levels of CEA and TD. The scoring system is as follows: 0 points for CEA(-) and TD(-); 1 point for CEA(+) and TD(-); 2 points for CEA(-) and TD(+); and 3 points for CEA(+) and TD(+). Patients were classified into four categories based on the score.

### Construction of the nomogram

A nomogram is a graphical representation of mathematical relationships, commonly used to estimate the results of a formula visually. In this study, a long-term survival prediction model for patients was developed based on the CEA-TD score combined with other clinical variables to assess the prognosis of colon cancer (CC) patients. Survival curves, calibration plots, concordance indices, and ROC curves were generated to evaluate the predictive ability of the nomogram for prognostic factors in both the training and validation cohorts.

### Statistical analysis

Statistical analyses were performed using SPSS software (version 25.0, Chicago, IL, USA) and GraphPad Prism 9. Categorical data are presented as frequencies (percentages), while continuous data are expressed as medians (interquartile ranges). The comparison of two categorical variables was performed using the chi-square test, Fisher’s exact test, or the Mann-Whitney U test, as appropriate. For comparisons among multiple groups, the Kruskal-Wallis test was applied. The Kaplan-Meier survival curve was used to assess overall survival in colon cancer patients, and the log-rank test was employed to compare differences between groups. A p-value of <0.05 was considered statistically significant.

## Results

### Demographic and clinical characteristics

**Figure 1:**
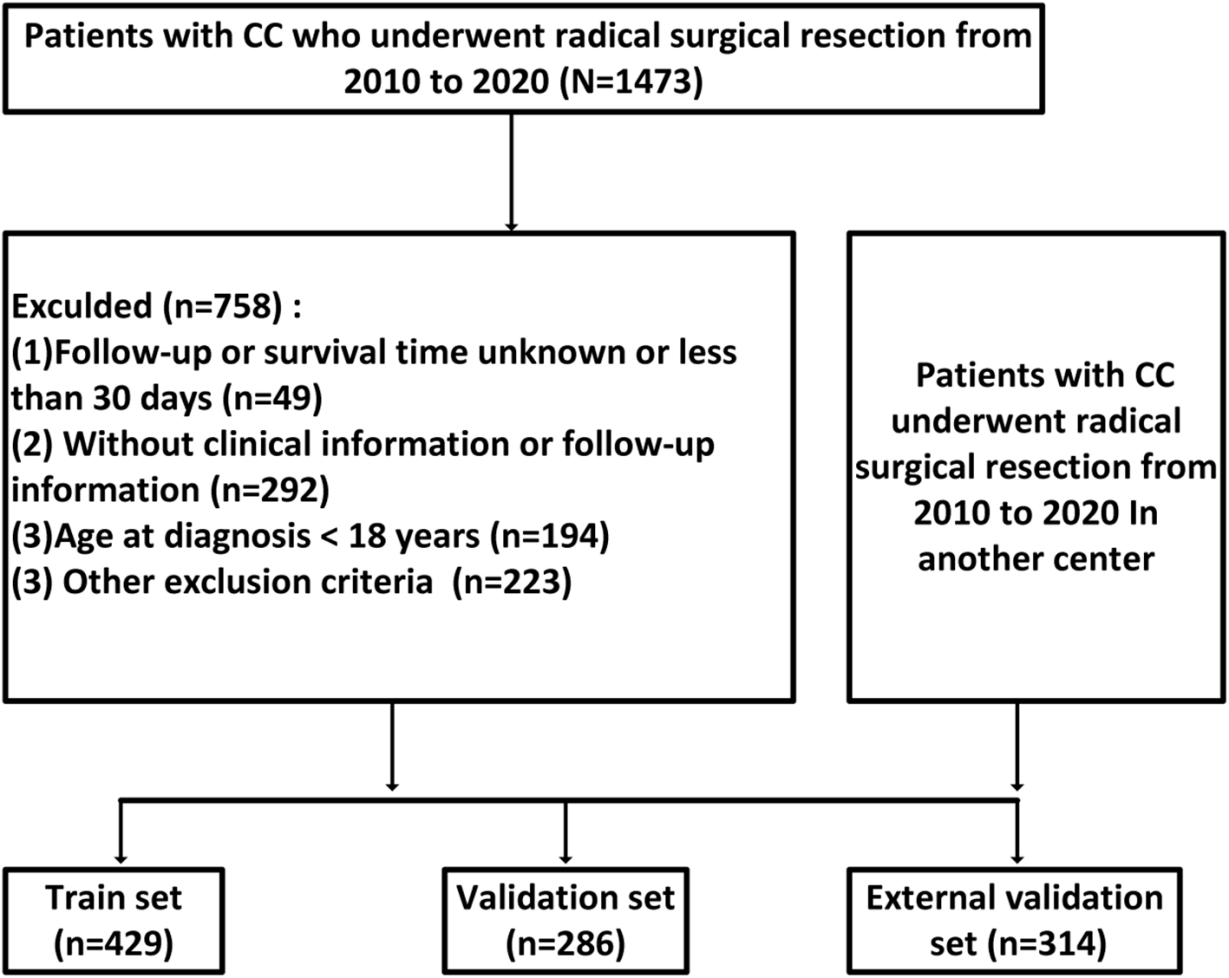
Patient cohort selection process flowchart.

A total of 1, 029 patients with locally advanced colon cancer were included in this study, comprising 715 patients from Hebei Provincial People’s Hospital and 314 patients from Shijiazhuang People’s Hospital (**Figure 1**). Of the 715 patients from Hebei Provincial People’s Hospital, 429 were randomly assigned to the training set and 286 to the validation set using a 60:40 ratio, based on the “caret” package in R software. The 314 patients from Shijiazhuang People’s Hospital were allocated to the external validation set. The proportions of TD(+) in the three cohorts were as follows: 15.9% (68/429) in the training set, 14.6% (42/286) in the validation set, and 16.9% (53/314) in the external validation set. The proportions of CEA positivity were 39.6% (170/429) in the training set, 40.6% (116/286) in the validation set, and 39.2% (123/314) in the external validation set. In the training cohort, there were 125 KRAS mutant, 205 wild-type, and 99 unknown cases; in the internal validation cohort, 80 mutant, 146 wild-type, and 60 unknown; and in the external validation cohort, 90 mutant, 158 wild-type, and 66 unknown. No significant differences in clinical factors were observed between the two validation sets and the training set (**Table 1** for further details).

**Table 1:** Baseline demographic and clinicopathological features of the patients.

Variable	Training set	Internal validation	External validation	P	
	(N = 429)	(N = 286)	(N = 314)	Train vs internal	Train vs external
Age (Years), Mean	53.4 (6.70)	53.5 (6.52)	49.2 (7.58)	0.921	0.297
Gender					
Female	188 (43.8%)	125 (43.7%)	141 (44.9%)	0.975	0.827
Male	241 (56.2%)	161 (56.3%)	173 (55.1%)		
Marital status					
Married	250 (58.3%)	177 (61.9%)	195 (62.1%)	0.375	0.329
Unmarried	179 (41.7%)	109 (38.1%)	119 (37.9%)		
CEA					
negative/normal	259 (60.4%)	170 (59.4%)	191 (60.8%)	0.864	0.961
positive/elevated	170 (39.6%)	116 (40.6%)	123 (39.2%)		
Primary tumor site					
Left	171 (39.9%)	113 (39.5%)	131 (41.7%)	0.988	0.664
Right	258 (60.1%)	173 (60.5%)	183 (58.3%)		
Tumor size(mm), Mean					
<5cm	268 (62.5%)	162 (56.6%)	174 (55.4%)	0.139	0.063
≥5cm	161 (37.5%)	124 (43.4%)	140 (44.6%)		
TNM stage					
I	81 (18.9%)	49 (17.1%)	54 (17.2%)	0.810	0.121
II	146 (34.0%)	102 (35.7%)	130 (41.4%)		
III	202 (47.1%)	135 (47.2%)	130 (41.4%)		
Grade					
I	74 (17.2%)	52 (18.2%)	50 (15.9%)	0.880	0.260
II	265 (61.8%)	178 (62.2%)	182 (58.0%)		
III	90 (21.0%)	56 (19.6%)	82 (26.1%)		
pT stage					
T1/2	109 (25.4%)	75 (26.2%)	64 (20.4%)	0.807	0.129
T3/4	320 (74.6%)	211 73.8%)	250 (79.6%)		
pN stage					
N0	227 (52.9%)	151 (52.8%)	184 (58.6%)	0.921	0.199
N1	133 (31.0%)	86 (30.1%)	79 (25.2%)		
N2	69 (16.1%)	49 (17.1%)	51 (16.2%)		
TD					
TD (-)	361 (84.1%)	244 (85.3%)	261 (83.1%)	0.751	0.784
TD (+)	68 (15.9%)	42 (14.7%)	53 (16.9%)		
CEA-TD score					
0	232 (54.1%)	157 (54.8%)	168 (53.5%)	0.795	0.958
1	129 (30.0%)	87 (30.5%)	93 (29.6%)		
2	27 (6.3%)	13 (4.6%)	23 (7.3%)		
3	41 (9.6%)	29 (10.1%)	30 (9.6%)		

A comparison of clinical and pathological features between TD(+) and TD(-) patients revealed that TD(+) patients had higher tumor stages, T stages, and a higher proportion of CEA positivity compared to TD(-) patients, with statistically significant differences (P<0.05). However, no significant differences were observed in relation to age, gender, tumor size, tumor location, or histological grade (**Table 2**).

**Table 2:** Baseline demographic and clinicopathological features of the patients with the presence or absence of TD.

Characteristics	All (N = 429)	TD (-) (N = 361)	TD (+) (N = 68)	P
Age (Years), Mean	53.5 (6.72)	52.8 (6.69)	53.7 (6.73)	0.281
Gender				
Female	188 (43.8%)	153 (42.4%)	35 (51.5%)	0.210
Male	241 (56.2%)	208 (57.6%)	33 (48.5%)	
Marital status				
Married	250 (58.3%)	207 (57.3%)	43 (63.2%)	0.441
Unmarried	179 (41.7%)	154 (42.7%)	25 (36.8%)	
CEA				
negative/normal	259 (60.4%)	232 (64.3%)	27 (39.7%)	<0.001
positive/elevated	170 (39.6%)	129 (35.7%)	41 (60.3%)	
Primary tumor site				
Left	171 (39.9%)	150 (41.6%)	21 (30.9%)	0.130
Right	258 (60.1%)	211 (58.4%)	47 (69.1%)	
Tumor size(mm), Mean	46.3 (24.7)	44.4 (23.8)	56.7 (27.0)	
<5cm	268 (62.5%)	231 (64.0%)	37 (54.4%)	0.174
≥5cm	161 (37.5%)	130 (36.0%)	31 (45.6%)	
Grade				
I	74 (17.2%)	69 (19.1%)	5 (7.4%)	0.059
II	265 (61.8%)	217 (60.1%)	48 (70.6%)	
III	90 (21.0%)	75 (20.8%)	15 (22.1%)	
Stage				
I	81 (18.9%)	81 (22.4%)	0 (0%)	<0.001
II	146 (34.0%)	146 (40.4%)	0 (0%)	
III	202 (47.1%)	134 (37.2%)	68 (100%)	
T				
T1-2	109 (25.4%)	103 (28.5%)	6 (8.8%)	0.001
T3-4	320 (74.6%)	258 (71.5%)	62 (91.2%)	
N				
N0	227 (52.9%)	227 (62.9%)	0 (0%)	<0.001
N1	133 (31.0%)	92 (25.5%)	41 (60.3%)	
N2	69 (16.1%)	42 (11.6%)	27 (39.7%)	
CEA-TD score				
0	232 (54.1%)	232 (64.3%)	0 (0%)	<0.001
1	129 (30.1%)	129 (35.7%)	0 (0%)	
2	27 (6.3%)	0 (0%)	27 (39.7%)	
3	41 (9.6%)	0 (0%)	41 (60.3%)	

### Identification of prognostic clinical factors for OS

Patients were categorized into four groups based on the CEA-TD score: 0 points (CEA(-), TD(-)), 1 point (CEA(+), TD(-)), 2 points (CEA(-), TD(+)), and 3 points (CEA(+), TD(+)). Univariate Cox regression analysis showed a significant association between survival time and the CEA-TD score. Additionally, the multivariate Cox regression analysis show that T stage, N stage, TNM stage, histological grade, and CEA-TD score were all significantly related to patient prognosis. **Table 3** provides the estimated regression coefficients and hazard ratios for each variable.

**Table 3:** Univariate and multivariate analysis of overall survival in the training cohort.

Variable	Univariate		Multivariate	
	HR (95% CI)	*P*	HR (95% CI)	*P*
Age(years)				
≤50	Reference			
>50	1.014 (0.988-1.041)	0.284		
Gender				
Female	Reference			
Male	0.977 (0.686-1.391)	0.897		
Tumor size				
<5cm	Reference			
≥5cm	1.392 (0.974-1.990)	0.069		
pT stage				
T1/2	Reference		Reference	
T3/4	5.139 (2.786-9.480)	<0.001	3.801 (2.000-7.226)	<0.001
pN stage				
N0	Reference		Reference	
N1	2.588 (1.699-3.941)	<0.001	1.679 (1.061-2.658)	0.027
N2	4.670 (2.970-7.343)	<0.001	2.680 (1.594-4.507)	<0.001
TNM stage				
I	Reference			
II	3.080 (1.442-6.582)	0.004		
III	6.921 (3.416-14.026)	<0.001		
Grade				
I	Reference		Reference	
II	2.964 (1.611-5.452)	<0.001	2.002 (1.046-3.831)	0.036
III	3.986 (2.041-7.784)	<0.001	2.996 (1.486-6.041)	0.002
Marital status				
Married	Reference			
Unmarried	1.237 (0.869-1.759)	0.238		
Primary tumor site				
Left	Reference		Reference	
Right	2.204 (1.472-3.301)	<0.001	2.257 (1.504-3.389)	<0.001
CEA				
negative/normal	Reference			
positive/elevated	2.437(1.608-3.692)	<0.001		
TD				
TD (-)	Reference			
TD (+)	4.264 (2.911-6.246)	<0.001		
CEA-TD score				
0	Reference			
1	3.397 (2.172-5.315)	<0.001	2.545 (1.612-4.017)	<0.001
2	5.729 (3.058-10.732)	<0.001	2.672 (1.372-5.204)	0.004
3	8.785 (5.240-14.728)	<0.001	4.284 (2.435-7.535)	<0.001

### Comparison of long-term prognosis in colon cancer patients

**Figure 2:**
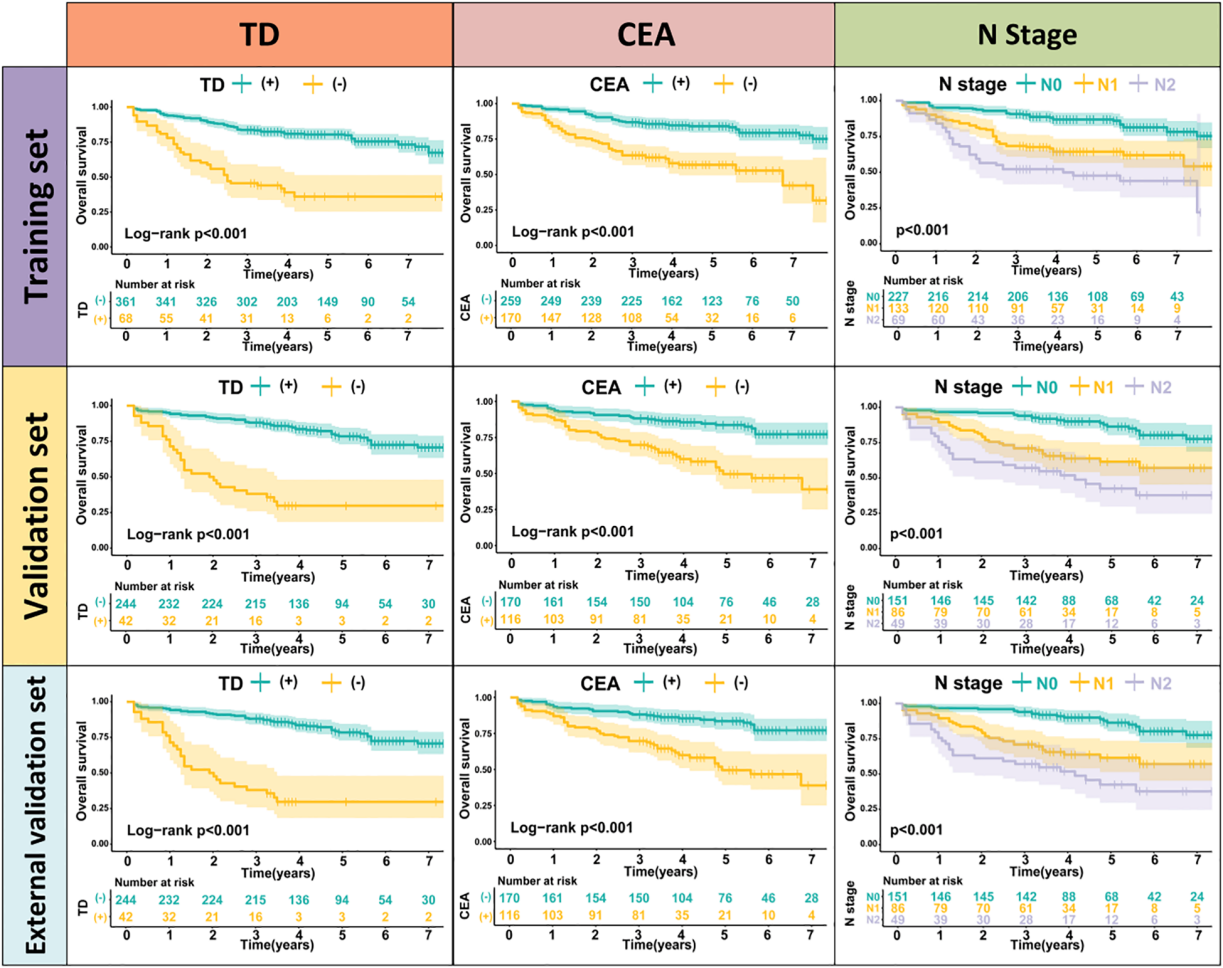
The Kaplan-Meier survival curves for predicting 1-, 3-, and 5-year OS in three sets.

**Figure 3:**
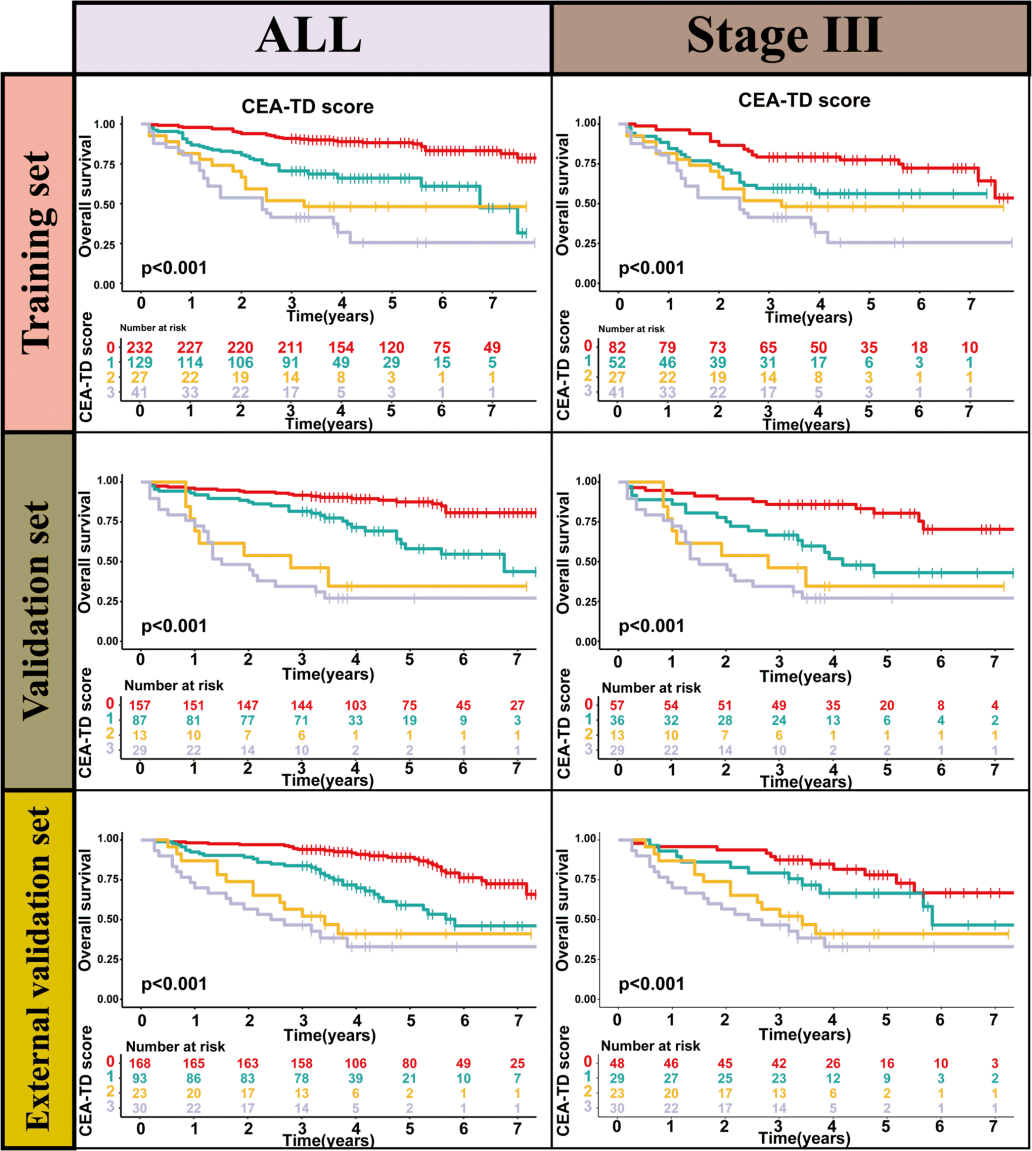
The Kaplan-Meier survival curves of CEA-TD score for predicting 1-, 3-, and 5-year OS in three sets.

**Figure 4:**
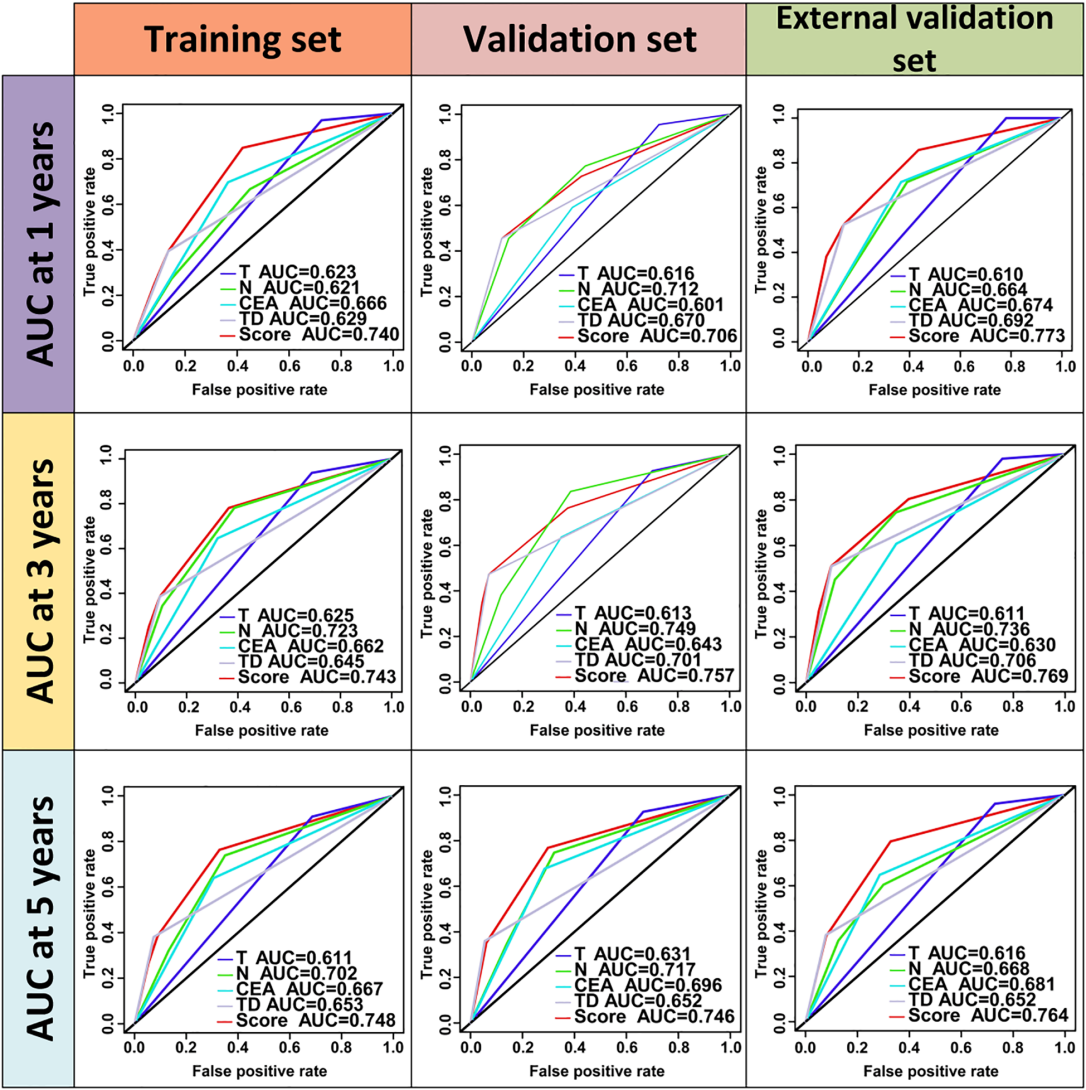
The ROC curves of CEA-TD score for predicting 1-, 3-, and 5-year OS in three sets.

TD(+) patients were significantly associated with poor OS compared to TD(-) patients (HR = 4.264, 95% CI: 2.911-6.246, P<0.001). Similarly, CEA(+) patients had significantly worse OS than CEA(-) patients (HR = 2.437, 95% CI: 1.608-3.692, P<0.001). In the N stage, patients with lymph node metastasis had worse prognoses compared to N0 patients (N1: HR = 2.588, 95% CI: 1.699-3.941, P<0.001; N2: HR = 4.670, 95% CI: 2.970-7.343, P<0.001) (**Figure 2**). When patients were grouped based on both TD and CEA, significant survival differences were observed among the four groups. Further, survival times also differed significantly among the four groups in Stage III patients (**Figure 3**, **Supplementary Table 1**). To further explore the prognostic ability of the CEA-TD score, the area under the curve (AUC) was plotted, as shown in **Figure 4**.

### Development and validation of the CEA-TD-based nomogram

**Figure 5:**
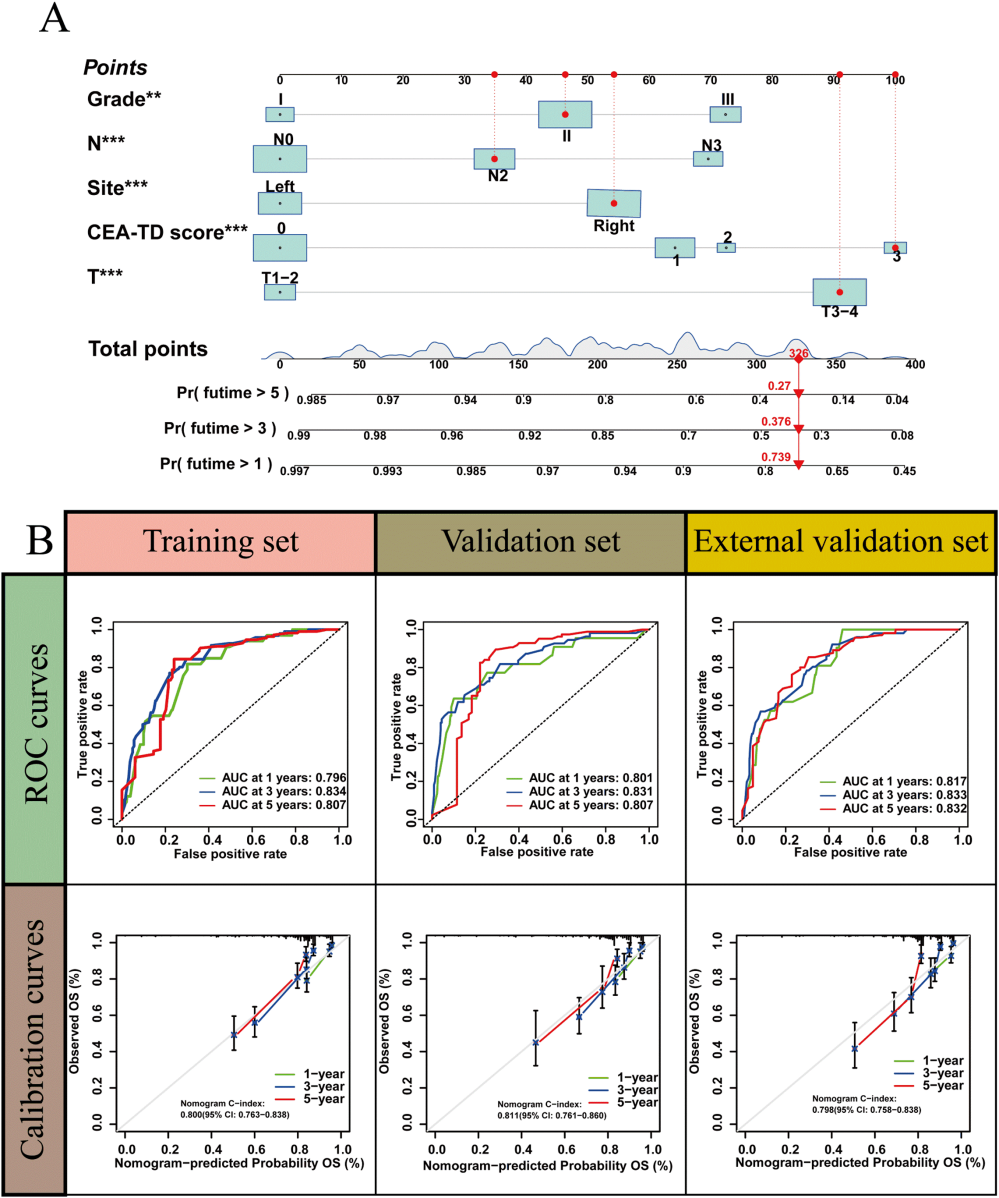
A nomogram based on CEA-TD score. **(A)** The nomogram was built based on five clinical variables in the training set. **(B)** The ROC curves, calibration curves for predicting OS at 1-, 3-, and 5-year overall survival.

**Figure 6:**
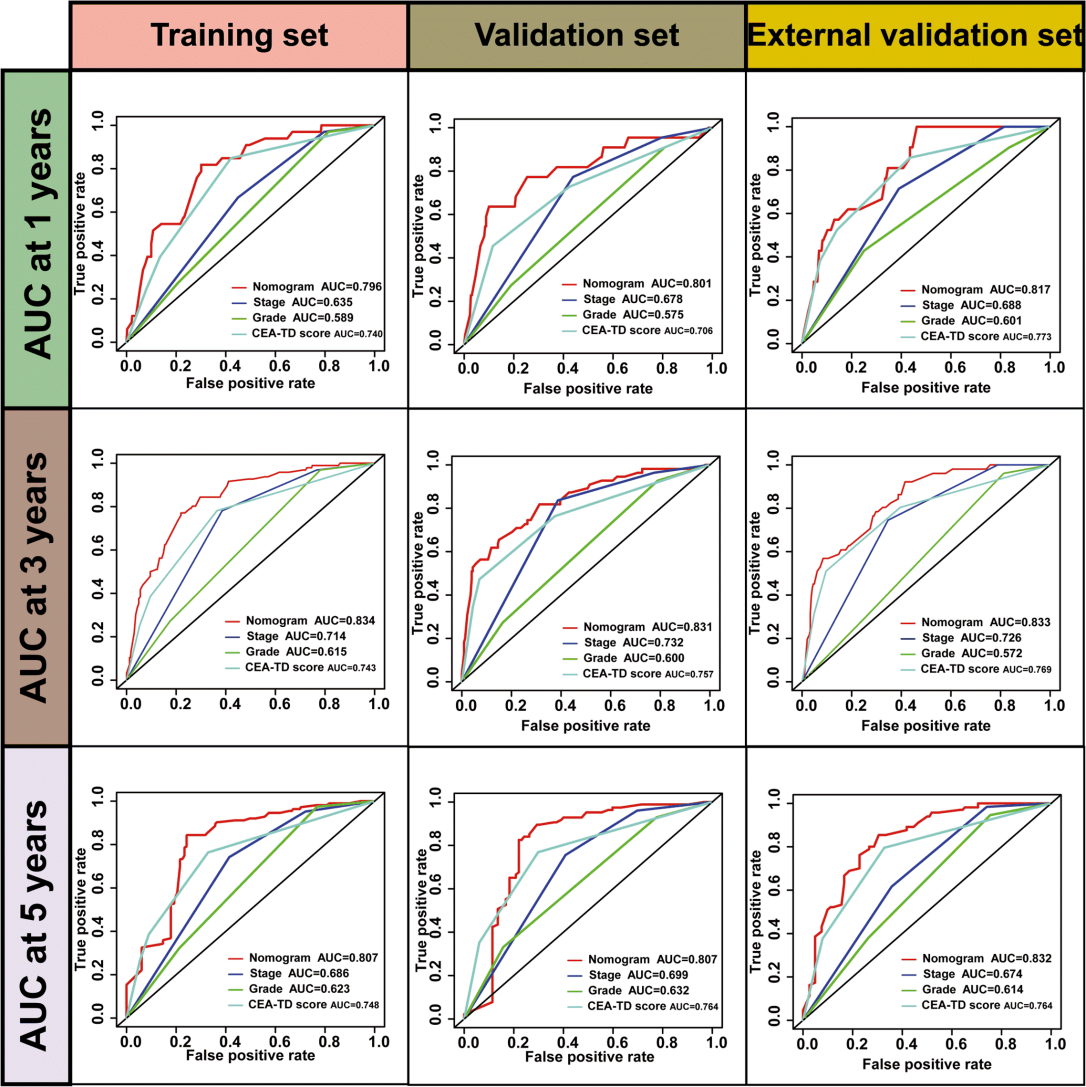
The ROC curves for valuating Nomogram performance.

**Figure 7:**
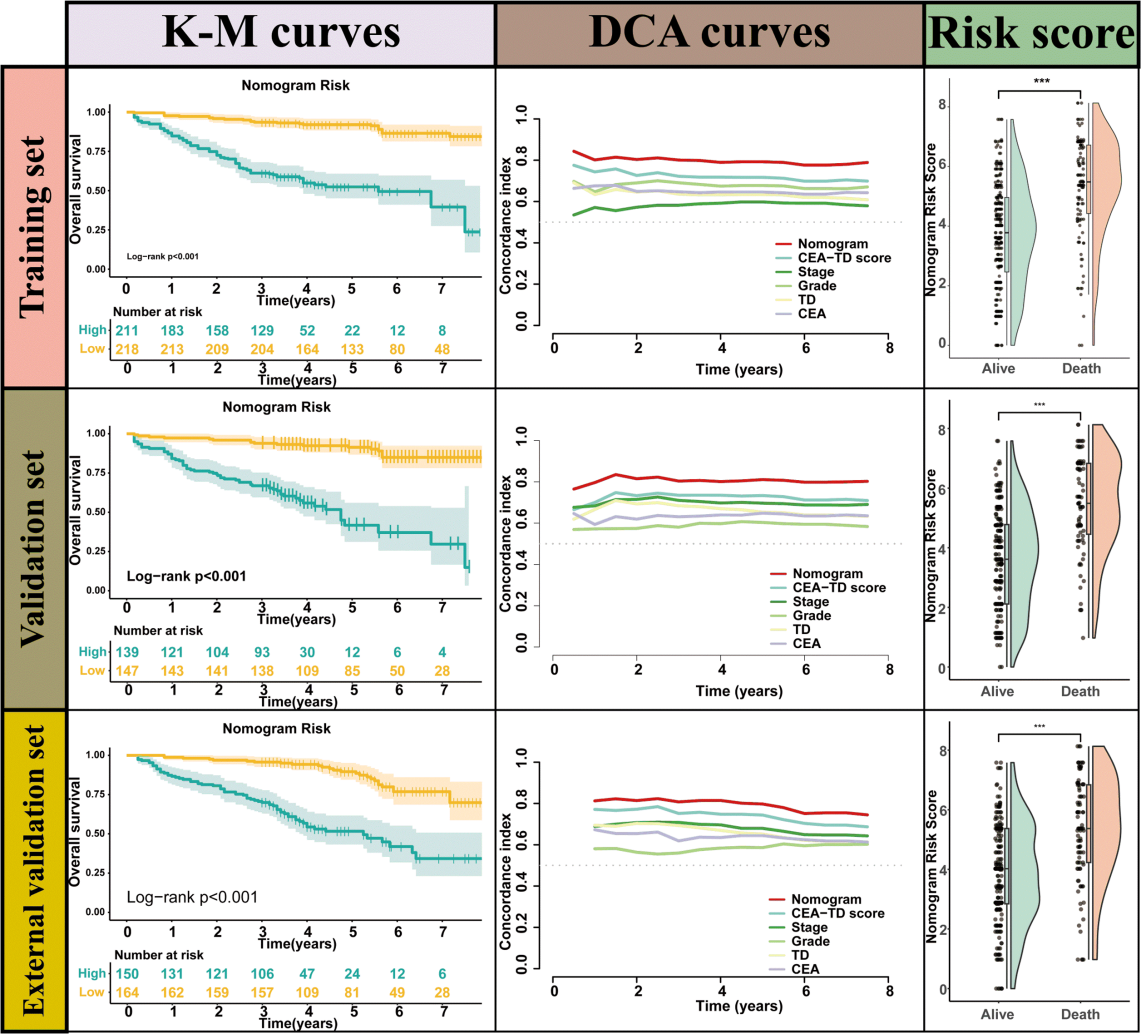
The Kaplan-Meier survival curves, time-dependent C-index curve and nomogram risk score curves for valuating Nomogram performance.

A new nomogram was developed to estimate the prognosis of colon cancer patients using the CEA-TD score combined with four additional clinical variables: T stage, N stage, histological grade, and tumor location (**Figure 5A**). In the nomogram, each variable is represented by a vertical scale indicating its value range. By aligning the variable values and observing the intersections on the nomogram, survival probabilities at 1, 3, and 5 years can be estimated. Subsequently, calibration curves, ROC curves, and time-dependent concordance index (C-index) curves were generated to assess the predictive performance of the nomogram, which demonstrated good applicability and accuracy. The AUC of the nomogram in the training set was 0.796 at 1 year, 0.834 at 3 years, and 0.807 at 5 years, outperforming other variables in the internal validation set, where the AUCs were 0.801 at 1 year, 0.831 at 3 years, and 0.807 at 5 years (**Figures 5B**, **6**). The C-index of the nomogram was 0.800 (95% CI: 0.763-30.838), also higher than other clinical variables (**Figure 7**). Moreover, patients were stratified into high-risk and low-risk groups based on the median risk score calculated from the nomogram. Patients in the high-risk group had shorter survival times compared to those in the low-risk group (**Figure 7**). The nomogram risk scores for patients with different survival statuses are shown in **Figure 7**, indicating that an increase in the risk score corresponds to a higher mortality rate in CC patients. Finally, risk scores derived from the same formula used in the nomogram were calculated for both the internal and external validation sets. In both validation cohorts, patients with low-risk scores had better prognoses than those with high-risk scores. These results suggest that the nomogram can accurately and conveniently predict the prognosis of LOCC patients.

### Preoperative and postoperative CEA analysis

Patients were classified into three groups based on preoperative and postoperative (4–6 weeks) serum CEA levels: normal both pre- and postoperatively, elevated preoperatively but normalized postoperatively, and persistently elevated. In the training cohort, the groups included 259, 92, and 78 patients, respectively; in the internal validation cohort, 170, 65, and 51; and in the external validation cohort, where postoperative CEA data were missing for 40 patients, the groups included 162, 63, and 49, respectively. Kaplan–Meier analyses showed significantly different survival among the three groups (p<0.001), with the best outcomes in patients with consistently normal CEA, intermediate outcomes in those with normalization postoperatively, and the worst outcomes in those with persistently elevated CEA (**Supplementary Figure 1**). These findings suggest that dynamic changes in CEA may more accurately reflect prognostic risk.

### Subgroup analysis

**Figure 8:**
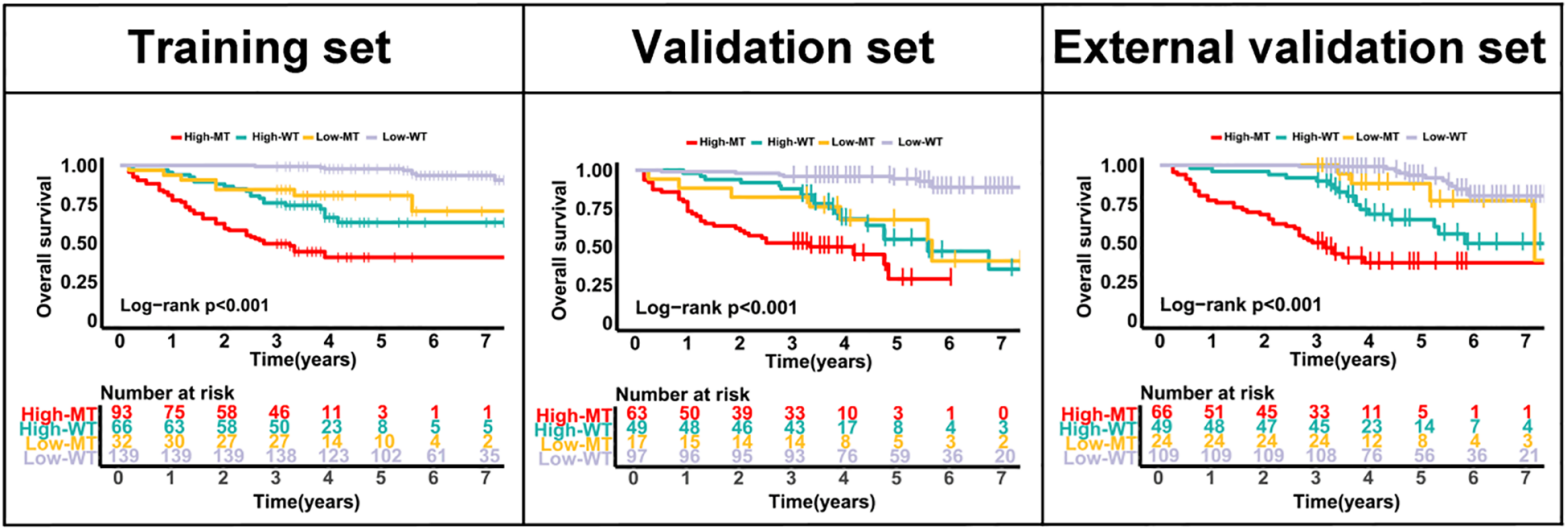
Kaplan–Meier curves for overall survival according to CEA-TD risk groups and KRAS status in the training, internal validation, and external validation cohorts.

Across all three cohorts, the model effectively stratified overall survival within both KRAS mutant and wild-type subgroups. High-risk patients consistently showed significantly worse outcomes than low-risk patients (all p<0.001), confirming the robustness of the CEA-TD based model irrespective of KRAS status (**Figure 8**).

## Discussion

This study presents a comprehensive model for predicting the long-term survival of colon cancer patients by integrating two prognostic factors, CEA and TD, along with other clinical variables. Our findings indicate that the combination of CEA and TD significantly enhances the prognostic accuracy compared to traditional methods, such as TNM staging, in predicting overall survival in colon cancer patients. We developed a CEA-TD scoring system and validated its predictive performance using a large cohort of 1, 029 patients from two hospitals. The nomogram, constructed based on this scoring system, demonstrated excellent predictive accuracy, with higher area under the curve values at 1, 3, and 5 years compared to individual clinical factors, underscoring its potential clinical utility. Moreover, the nomogram’s concordance index further validated its effectiveness, as it outperformed other clinical variables in predicting patient prognosis. These results suggest that combining CEA and TD with other significant clinical markers, such as T stage, N stage, and histological grade, offers a promising and clinically relevant tool for prognosis prediction in colon cancer patients.

When compared to previous studies, our research contributes novel insights into the integration of CEA and TD as a combined prognostic tool for colon cancer. CEA has long been recognized as a critical serum marker for colorectal cancer, particularly for monitoring recurrence and assessing treatment response (21, 22). Numerous studies have examined the prognostic value of CEA in colon cancer, with results showing that elevated CEA levels are associated with poorer prognosis and higher risk of recurrence (23–25). However, the prognostic accuracy of CEA alone is often limited, particularly in early-stage cancers or localized disease. Our study supports this limitation and demonstrates that combining CEA with TD further enhances survival prediction. This finding aligns with a study by Leonardo et al, who found that tumor deposits were an independent prognostic factor in colon cancer, particularly in patients with stage III disease (26). Their study, which explored the clinical significance of TD in CRC, suggests that TD is a marker of aggressive tumor behavior, similar to our findings.

In contrast to earlier studies that focused primarily on individual markers such as TNM staging, our study incorporates a combination of clinical factors, which has shown to improve prognostic prediction. Previous work by Lea et al. demonstrated the prognostic utility of the TNM staging system, which is currently the gold standard for colon cancer prognosis (27). However, several studies have highlighted its limitations in predicting outcomes, particularly in patients with locally advanced colon cancer. For instance, studies have shown that TNM staging does not fully account for the heterogeneity within patient populations, with some patients having worse outcomes despite similar staging (28). This study addresses such limitations by combining TD and CEA with TNM staging, which allows for a more nuanced risk stratification and better prediction of long-term survival. Additionally, the integration of CEA and TD into a nomogram represents a shift toward personalized medicine, moving beyond a one-size-fits-all approach and providing individualized treatment recommendations based on a patient’s specific risk profile. MSI status and ctDNA have been increasingly recognized as powerful predictors of recurrence and treatment response, offering valuable biological insights into tumor behavior. However, their widespread adoption in routine clinical practice remains limited by issues of accessibility, cost, and the need for specialized laboratory techniques. In contrast, CEA and TD are routinely assessed in standard clinical and pathological workflows, making the CEA–TD score highly feasible and cost-effective for real-world application. Compared to the model developed by, Zheng et al. (29) our model demonstrates superior performance, with a C-index of 0.800, which is higher than the 0.727 reported in their study. In addition to the differences in model performance, our cohort included patients with locally advanced colon cancer, rather than being limited to stage III colon cancer.

From a molecular mechanism perspective, the presence of tumor deposits in colon cancer reflects the tumor’s invasive and metastatic potential, suggesting a more aggressive biological phenotype. Tumor deposits are considered a form of extramural spread, where tumor cells or clusters are found outside the primary tumor site, often in peritoneal or lymphatic tissues. These deposits may indicate early metastatic dissemination, even in the absence of clinically detectable distant metastasis, and their presence has been associated with a poor prognosis (30). Recent molecular studies have suggested that TDs are linked to epithelial-mesenchymal transition, a process that plays a pivotal role in tumor invasion and metastasis. Elevated CEA levels have also been associated with EMT and increased metastatic potential in various cancers, including colorectal cancer (31). Our findings support this, as we observed that TD(+) patients had higher tumor stages and a greater proportion of CEA positivity, further suggesting that these markers are reflective of the underlying biological aggressiveness of the tumors. Thus, the CEA-TD score serves not only as a prognostic tool but also as an indicator of the tumor’s biological behavior, shedding light on the molecular underpinnings of colon cancer progression.

Despite the promising results, this study has several limitations that warrant consideration. First, while the external validation cohort strengthens the generalizability of our findings, the study remains retrospective, and the risk of selection bias cannot be fully excluded. And the cohort was derived from a single region with relatively homogeneous ethnic characteristics, which may limit the generalizability of our findings to broader populations. Further prospective studies would be essential to validate the predictive accuracy of the nomogram in different patient populations and clinical settings. Additionally, while we included a broad range of clinical variables in our analysis, there are other potential prognostic markers, such as molecular subtypes of colorectal cancer, that could enhance the predictive accuracy of the nomogram. Incorporating these additional markers, along with a more detailed molecular characterization of tumor deposits, could further improve the model’s ability to predict long-term survival. Moreover, in our study did not account for detailed information on adjuvant therapy or patient comorbidities, which may independently affect prognosis and represent a limitation. Future prospective studies should incorporate these factors to further refine and validate the predictive model.Future research should focus on incorporating these factors to refine the nomogram and assess its applicability in patients who have undergone different treatment regimens.

In conclusion, this study successfully integrates CEA and tumor deposits with other clinical variables to develop a novel prognostic tool for predicting long-term survival in colon cancer patients. The CEA-TD-based nomogram demonstrated superior predictive performance compared to traditional staging systems and individual biomarkers, providing an effective and clinically relevant method for risk stratification. This tool could aid clinicians in making more informed decisions regarding treatment strategies, particularly for patients with locally advanced colon cancer. The findings of this study contribute to the growing body of literature on personalized medicine in colorectal cancer, emphasizing the importance of integrating multiple prognostic factors to enhance survival prediction and improve patient outcomes. Future studies, particularly those that incorporate molecular subtypes and additional treatment variables, are needed to further validate and refine this predictive model.

## Data Availability

The original contributions presented in the study are included in the article/**Supplementary Material**. Further inquiries can be directed to the corresponding author.

## Supplementary material

Supplementary Figure 1 Kaplan–Meier curves of overall survival stratified by postoperative CEA status in the training, internal validation, and external validation cohorts.

Supplementary Table 1 Cox Regression Analysis Results for CEA-TD score subgroup Comparisons.
