# Supplementary material for: A large-scale study integrating CEA and tumor deposits to evaluate prognosis in colon cancer patients
Source: Front Oncol. 2025 Dec 17;15:1626538. doi: 10.3389/fonc.2025.1626538 (PMC12754172; doi:10.3389/fonc.2025.1626538)
Supplement: Supplementary Figure 1 — Kaplan–Meier curves of overall survival stratified by postoperative CEA status in the training, internal validation, and external validation cohorts. [file DataSheet1.docx]

Supplementary Table 1 Cox Regression Analysis Results for **CEA-TD score subg**roup Comparisons

| **Variable** | **Training cohort** | | **Internal validation** | | **External validation** | |
| --- | --- | --- | --- | --- | --- | --- |
| **CEA-TD score** | **HR (95% CI)** | ***P*** | **HR (95% CI)** | ***P*** | **HR (95% CI)** | ***P*** |
| 0 vs 1 | 3.507 (2.234-5.506) | <0.001 | 3.023 (1.743-5.243) | <0.001 | 3.019 (1.835-4.965) | <0.001 |
| 0 vs 2 | 2.399 (1.749-3.290) | <0.001 | 2.723 (1.799-4.122) | <0.001 | 2.565 (1.832-3.592) | <0.001 |
| 0 vs 3 | 2.093 (1.756-2.494) | <0.001 | 2.162 (1.757-2.659) | <0.001 | 2.043 (1.670-2.499) | <0.001 |
| 1 vs 2 | 1.688 (0.928-3.070) | 0.0827 | 2.786 (1.260-6.157) | 0.008 | 2.138 (1.124-4.067) | 0.018 |
| 1 vs 3 | 1.593 (1.254-2.023) | <0.001 | 1.981 (1.485-2.643) | <0.001 | 1.706 (1.283-2.267) | <0.001 |
| 2 vs 3 | 1.490 (0.780-2.849) | 0.224 | 1.329 (0.588-3.006) | 0.493 | 1.347 (0.665-2.730) | 0.407 |


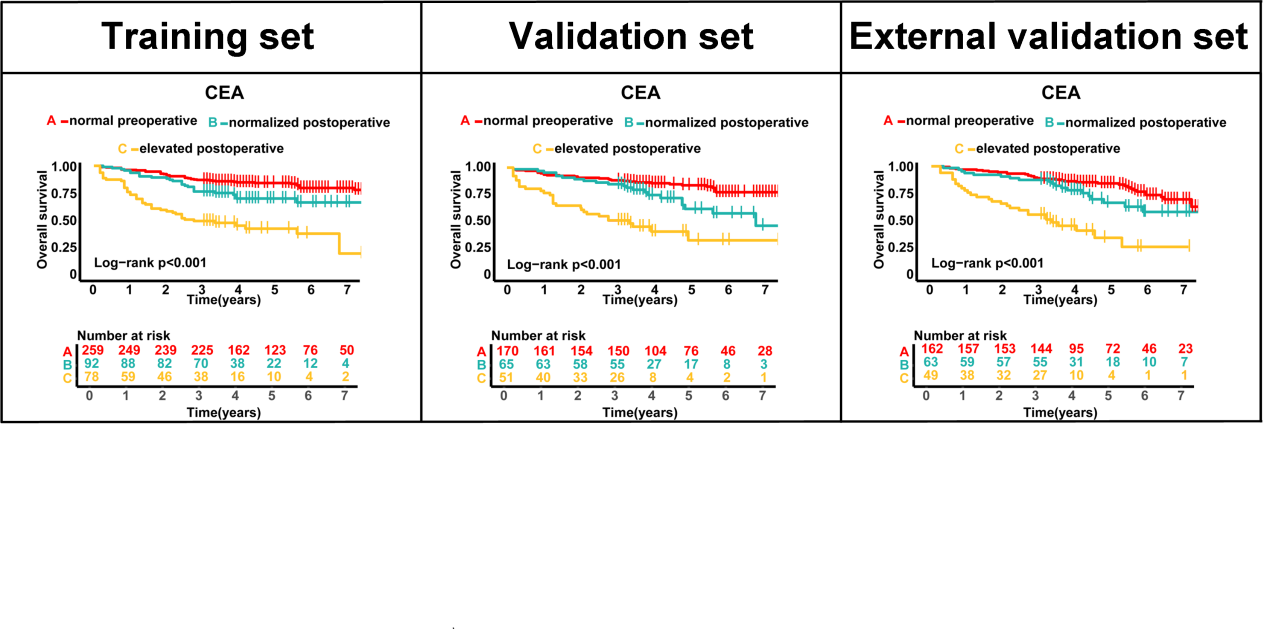


Supplementary Figure 1. Kaplan–Meier curves of overall survival stratified by postoperative CEA status in the training, internal validation, and external validation cohorts.
